# Somatic distress among Syrian refugees with residence permission in Germany: analysis of a cross-sectional register-based study

**DOI:** 10.1186/s12889-021-10731-x

**Published:** 2021-05-12

**Authors:** Andrea Borho, Eva Morawa, Gregor Martin Schmitt, Yesim Erim

**Affiliations:** 1 Department of Psychosomatic Medicine and Psychotherapy, Friedrich-Alexander University Erlangen-Nürnberg (FAU), Erlangen, Germany; 2 Erlangen City Council, Job Center, Erlangen, Germany

**Keywords:** Somatic distress, Somatic symptoms, Mental health, Syrian refugees, Health care utilization, Comorbidities, Germany

## Abstract

**Background:**

Previous studies have already proven high rates of common mental disorders in Syrian refugees. Nevertheless, little is known about the patterns of somatic distress among this refugee population. For this reason, we aimed to examine the prevalence, co-occurrence, and risk factors of somatic distress among Syrian refugees in Germany.

**Methods:**

This study analyzes the second measurement point (*N* = 116) of a prospective register-based survey among 200 adult Syrian refugees with residence permission in Germany. The survey consisted of information on sociodemographic and migration-specific characteristics, health care utilization, traumatic life events, acculturative stress (Barcelona Immigration Stress Scale (BISS); subscales: perceived discrimination, intercultural contact stress, homesickness, and general psychosocial stress), and self-reported outcomes of somatic distress (Patient Health Questionnaire (PHQ-15)), depression (PHQ-9), generalized anxiety disorder (GAD-7), and post-traumatic symptoms (Essen Trauma Inventory (ETI)).

**Results:**

Almost half of the respondents (49.1%) were identified as being at risk of somatic distress (PHQ-15 score ≥ 6), and even 24.1% being bothered by moderate-to-severe levels of somatic distress (PHQ-15 score ≥ 10). The most robust associations with somatic distress were found for female gender, the amount of health care utilization, multiple trauma exposures, general psychosocial stress, and self-reported depression and anxiety symptoms. High comorbidities with somatic distress were shown for all of the common mental disorders studied.

**Conclusions:**

The presented study reveals a significant risk of somatic distress among this displaced population and highlights implications for policy and health care providers.

**Supplementary Information:**

The online version contains supplementary material available at 10.1186/s12889-021-10731-x.

## Background

In 2011, peaceful protests in Syria were violently suppressed, followed by 10 years of civil war and one of the largest refugee crises seen across the world to date. More than half of the Syrian population had to leave their homes and build a new life in another place or country [1]. As a result, since 2014, Syrians represent the largest group of refugees in Germany, with the highest number of asylum applications in 2016 (266,250 Syrian asylum seekers) [2].

It is now common knowledge that this population – who has been forced to flee war and armed conflict zones – is likely to have experienced traumatic events, including violence, loss of property, and separation from family [3–5]. Ongoing post-migration stressors in host countries, such as uncertainty about the extension of residence permission, language barriers, and perceived discrimination, can place additional strain on refugees’ mental health [3, 6, 7]. In this respect, high prevalence rates of mental disorders among Syrian refugees have been widely reported for posttraumatic stress disorder (PTSD; 11–84%), depression (15–65%), and anxiety disorders (14–60%) [3–5, 8–14].

These three common mental disorders have attracted the most attention in existing studies, while only a few researchers having investigated the prevalence and properties of somatic symptoms, including somatic distress (SOD), among this displaced population. Indeed, previous studies among refugee populations have shown rates of SOD as high as 63% [15–17]. An exception regarding the investigation of the Syrian refugee population represents a recent study among Syrian refugees, registered in Istanbul, Turkey. In this sample, 41.7% were at risk of SOD [18]. Since somatic symptoms are often the reason why people with mental health problems present to medical services first, not only should organic physical complaints be considered in order to increase health service efficiency, but also emotional complaints that express themselves physically [15, 19].

SOD, which is known as somatic symptom disorder under the DSM-V, is typically characterized by an extreme focus on physical symptoms that causes major emotional distress and problems in functioning, thereby increasing the perceived need for health care service utilization [20, 21]. It manifests as physical discomfort that may or may not be associated with another medical condition, producing symptoms such as insomnia or pain in the chest, head, or stomach. In addition to the associated burden of those affected, providing necessary treatment for this disorder poses a problem for mental health care institutions, especially in the case of refugees [15, 22, 23]. Somatization, the associated unconscious process by which psychological distress is expressed as physical symptoms, is particularly challenging in multicultural contexts, where patients and physicians often disagree about their illness-related beliefs and practices, treatments, and health care expectations [24]. Due to high levels of stigma concerning the use of mental health services, many refugee patients from non-Western countries, which include Syrians, expect to be predominantly treated for their bodily symptoms rather than for their mental problems [15, 18]. Social, cultural, and linguistic differences (e.g., the ambiguous meaning of the word heartache) between patients and physicians can create additional misunderstandings and barriers to the development of a therapeutic relationship [15].

By now, there is striking evidence about a somatic comorbidity among refugees with PTSD, depression, and anxiety disorders [16, 18, 25, 26]. Especially for refugees with depression or PTSD, high comorbidity rates with physical complaints have been reported [16, 27, 28]. A recently conducted epidemiological study of 502 asylum-seekers in Germany assessed mental disorders including somatization. Among this sample with 10% coming from Syria, 49.7% of the respondents screened positive for at least one of the mental disorders investigated, with 31% suffering from somatization, 21.7% from depression, and 34.9% from PTSD. In this study, 44.9% of the participants who screened positive for somatization had comorbid depression and as many as 60.3% had comorbid PTSD [16]. These findings suggest that somatization may be an important aspect of trauma, one of the main causes of emotional distress [29]. Evidence confirming this assumption was found in several refugee samples, reporting a positive correlation between somatization and the number of previously experienced different trauma types (e.g., violence, war experiences, natural disasters) [23, 30, 31].

In addition to these findings, researchers have shown that being female, being older, and having low language proficiency, low economic status, and low education levels are significant and common sociodemographic risk factors for SOD among refugees [18, 31–33]. However, factors that have hardly been considered in studies of SOD are acculturative stressors such as discrimination or homesickness. There is little research demonstrating that post-migration stress is associated with somatic symptom severity in refugee samples (e.g., traumatized refugees in Switzerland or Burmese refugees in Australia) [17, 34, 35].

Syrian refugees represent the largest refugee population in Germany and have already been subject of several studies regarding common mental disorders, except for SOD [9, 13, 36, 37]. A qualitative study by our research group on Syrian refugees provided evidence of somatization in half of the participants [38]. Based on this finding, we expected that SOD occurs frequently in this refugee population. To the best of our knowledge, thus far, little is known about the specific patterns of SOD among this displaced population in Germany. In particular, the association with post-migration stressors has hardly been examined.

Therefore, the central objective of the present study was to use a register-based approach to report the prevalence rates of SOD among Syrian refugees with permission to reside in Germany. The additional objectives were to examine their health care utilization, to analyze the frequency of single somatic symptoms, to reveal comorbidities with other common mental disorders, and to explore the association between socio-demographic, migration-specific and health variables, post-migration stressors, and common mental disorders, and the severity of somatic symptoms.

## Methods

### Data collection and study sample

Between July and December 2017, our research team contacted all Syrian refugees registered at the Job Center Erlangen (a city in Germany) to participate in the first measurement point of a longitudinal prospective study on mental health and integration of Syrian refugees in Germany (PROSREF) [39]. The Job Center is a German authority where refugees with residence permission are registered to receive social benefits from the government. Of the 518 Syrians registered at that time, 200 (38.61%) took part. Approximately 1.5 years later, in 2019, these 200 participants were invited to take part in the second measurement point. The presented analysis was carried out as cross-sectional register-based survey within the second measurement point of PROSREF.

Between February and April 2019, the cooperating Job Center Erlangen informed all Syrian refugees who had already participated in the first measurement point and were still registered at the Job Center about the next measurement point by post. For data protection reasons, unregistered refugees could not be contacted, as no contact details were available for them. They were invited to attend voluntarily in pre-arranged meetings in a room at the Erlangen City Hall. Of the initial 200 participants, 108 (54%) still took part and another eight Syrian refugees showed up together with some of the invited participants. Thus, 116 Syrian refugees formed the total study sample for the presented analysis. All participants fulfilled the following criteria: Arrival in Germany after 2014, residence in the city of Erlangen, possession of German residence permission, aged above 18, registration at the Job Center Erlangen, agreement to participate, and a good knowledge of the Arabic language (at least spoken).

All study materials, including information about the study, declaration of consent, privacy policy, and questionnaires, were provided to the participants in Arabic. However, thirteen of the 116 participants (11.2%) could not complete the questionnaire on their own and asked for help from an Arabic-speaking study team member. After completing the questionnaires, all participants received a shopping voucher of € 20 as remuneration.

Full details of the data collection and study sample selection, as well as the non-responder analyses, can be found in earlier publications [9, 39].

### Instruments

Well-established, validated self-evaluation instruments assessing acculturative stress and mental health problems, as well as a self-developed demographic, migration-specific, and health related questionnaire, were used for this survey. All questionnaires were translated by independent translators from German into Arabic, back-translated, and combined into a final version to help ensure reliability, validity, and appropriateness for the study population [9, 40].

#### Sociodemographic, migration-specific, and health related variables

This questionnaire captured information relating to the refugees’ sociodemographic and migration-specific characteristics, such as duration of stay in Germany or future validity period of the residence permit in Germany, and information on their health status and medical service utilization.

#### Acculturative stress

Acculturative stress was measured using the Barcelona Immigration Stress Scale (BISS) [41]. The BISS is a 42-item self-evaluation instrument that measures immigration-related stress. The instrument consists of four subscales: Perceived discrimination, intercultural contact stress (e.g., difficulties in the practice of religion), homesickness, and general psychosocial stress (e.g., financial problems) [42]. The items are responded to on a four-point Likert-type scale from 1 (totally disagree) to 4 (totally agree). Higher scores indicate higher levels of acculturative stress. The internal consistency in our sample was measured using Cronbach’s alpha and was shown to be very good (α = 0.95).

#### Somatic distress

SOD was assessed by the valid and reliable somatic symptom module of the Patient Health Questionnaire (PHQ-15) [43]. This somatic symptom severity scale inquires about 15 somatic symptoms that account for more than 90% of the physical complaints (excluding upper respiratory tract symptoms) reported in outpatient settings [44]. One of the items, namely, asking about menstrual pain and problems, was only meant for female participants. Subjects are asked how much each of the 15 symptoms bother them on a three-point Likert scale ranging from 0 (not bothered at all), over 1 (bothered a little), to 2 (bothered a lot), during the past 4 weeks. Thus, the total score ranges from 0 to 30, with higher scores indicating greater severity of somatic symptoms. The total score can be classified into one of four levels of SOD, i.e., minimal (score ≤ 4), mild (score ≥ 5), moderate (score ≥ 10), and severe (score ≥ 15) [45]. Herein, a cut-off score of 10 was adopted to separate participants with high or low SOD, since this was identified as optimal for predicting the diagnosis of somatoform disorder [46]. We also added a cut-off score of 6 to identify participants at risk of SOD [18, 32, 47]. Furthermore, participants were asked how much these symptoms caused difficulties in doing their work, managing their household, and interacting with people on a four-point scale from 0 (not difficult at all) to 3 (very difficult). The internal consistency in this sample was good (Cronbach’s α = 0.88).

#### Depressive symptoms

The severity of depressive symptoms was measured by the self-administered nine item depression subscale of the Patient Health Questionnaire (PHQ-9) [48], which assesses the presence of the nine DSM-IV criteria for major depression within the last 2 weeks. PHQ-9 scores range from 0 to 27, with scores of 10 or higher indicating clinically relevant depressive symptoms. Cronbach’s alpha in this sample was 0.87.

#### Anxiety symptoms

The seven-item Generalized Anxiety Disorder Scale (GAD-7) is a self-rating screening tool and severity measure of various anxiety symptoms [49]. Scores range from 0 to 21, with higher scores representing higher levels of anxiety. The cut-off for clinically relevant anxiety symptoms is a score of 10 or higher. Cronbach’s α in this sample was 0.92.

#### Traumatic events and PTSD

The Essen Trauma Inventory (ETI; Arabic version) captures experienced traumatic events and posttraumatic symptomatology according to DSM-IV [50]. This self-evaluation questionnaire assesses the type and amount of experienced traumatic events using a trauma list consisting of 14 potentially traumatic events, such as war experience, as well as an open category. For the worst traumatic event experienced, the participants answer questions about objective and subjective danger to life (A1 and A2 criteria). The scale also includes a 17-item PTSD symptom list covering intrusion, avoidance, and hyperarousal. Clinically apparent PTSD is indicated if a participant has experienced at least one traumatic event, meets criteria A1 and A2, and if the total score for the PTSD symptom list is ≥ 27. The Cronbach’s alpha in the present study sample was 0.95.

### Statistical analysis

All data analyses were carried out by means of IBM SPSS Statistics 24 (IBM Corporation, Armonk, New York, USA). Missing records in the questionnaires were replaced by the expectation maximization method (maximum 20% missing data accepted). For descriptive statistics, we depicted the means, standard deviations, ranges and frequencies. The prevalence rates for mental disorders were calculated based on the established cut-off scores. In order to equalize the conditions of the PHQ-15 questionnaire for differences in gender, we additionally calculated a sum score excluding the menstruation item. The differences between groups (men/women and, low/high SOD) were analyzed using *t*-tests for continuous variables and Chi-squared or Fisher’s exact tests for categorical data. An analysis of covariance was calculated to investigate the differences in the degree of SOD in relation to gender and controlling for age. Pearson’s and Spearman’s correlation coefficients were computed to examine the correlations between SOD and specific variables and instruments. After checking the required assumptions, multiple linear block wise regression analyses were calculated to explore the association between sociodemographic characteristics, migration-specific characteristics, health care utilization, and specific mental disorders, and the severity of somatic symptoms. In order to measure the effect sizes, we computed Cohen’s d and Cramer’s V. For Cohen’s d, small effect sizes can be assumed if d ≥ 0.2, medium effect sizes if d ≥ 0.5, and large effect sizes if d ≥ 0.8. For Cramer’s V, V ≥ 0.1 = small effect size, V ≥ 0.3 = medium effect size, and V ≥ 0.5 = large effect size [51]. A level of significance was predetermined at *p* ≤ 0.05 for all analyses.

## Results

### Sample characteristics

The present study sample consisted of 116 Syrian refugees (31.0% women) living in Germany for an average of 41.51 months (3.5 years). Their average age at the time of the survey was 36.7 years (SD = 10.9; range: 19–64 years). Table 1 shows the socio-demographic and migration-specific characteristics of the total study sample, as well as the stratification by gender and SOD severity. There were no significant differences in the sociodemographic and migration-specific characteristics between participants with low and high SOD, except for the amount of different types of traumatic experiences and acculturative stress. Participants with high SOD reported significantly more experienced trauma types (M = 3.82, SD = 3.09) than participants with low SOD (M = 2.59, SD = 2.34; *t* = − 1.97, *p* = 0.025, d = 0.485). Furthermore, the mean value of experienced acculturative stress was 1.88 (SD = 0.59; range: 1–4), indicating ratings oscillating between totally disagree and moderately disagree. The ratings of both homesickness and general psychosocial stress ranged between moderately disagree and moderately agree. Intercultural contact stress and perceived discrimination were both rated lower than the mean acculturative stress score. Participants in the high SOD group differed significantly from the participants in the low SOD group in terms of general psychosocial stress (high SOD: M = 2.42, SD = 0.59; low SOD: M = 2.04, SD = 0.72; *t* = − 2.57, *p* = 0.012, d = 0.550) and homesickness (high SOD: M = 2.79, SD = 0.71; low SOD: M = 2.33, SD = 0.81; *t* = − 2.68, *p* = 0.008, d = 0.584).

**Table 1:** Sociodemographic and migration-specific characteristics of the total sample and stratification by gender and somatic distress severity (*N* = 116)

	**Total (*N*** **= 116)**		**Male (*n*** **= 80)**		**Female (*n*** **= 36)**		**Low somatic distress**^**1**^ **(*n*** **= 88)**		**High somatic distress**^**2**^ **(*n*** **= 28)**	
	n	%^a^*	n	%^a^*	n	%^a^*	n	%^a^*	n	%^a^*
**Age group**										
18–24	16	13.8	14	17.5	2	5.6	15	17.0	1	3.6
25–44	75	64.7	52	65.0	23	63.9	54	61.4	21	75.0
≥ 45	25	21.6	14	17.5	11	30.6	19	21.6	6	21.4
**Marital status**										
Single	34	29.3	34	42.5	0	0.0	32	36.4	2	7.1
Married/in a relationship	77	66.4	45	56.3	32	88.9	53	60.2	24	85.7
Divorced/Widowed	5	4.3	1	1.3	4	11.1	3	3.4	2	7.1
**Current activities**^**b**^										
School/Studies	17	14.7	15	18.8	2	5.6	15	17.0	2	7.1
Employment^c^	47	40.5	40	50.0	7	19.4	37	42.0	10	35.7
**Family in crisis areas**^**d**^	89	76.7	61	76.3	28	77.8	65	73.9	24	85.7
	**M** **(SD)**	**Range**	**M** **(SD)**	**Range**	**M** **(SD)**	**Range**	**M** **(SD)**	**Range**	**M** **(SD)**	**Range**
**Education in years** (*n* = 115)	10.80 (5.47)	0–30	11.51 (5.28)	0–30	9.25 (5.62)	0–20	10.55 (5.87)	0–30	11.57 (3.40)	6–20
**Duration of stay in Germany**^**e**^ (*n =* 114)	41.51 (7.33)	9–67	42.27 (6.90)	9–67	39.80 (8.12)	18–67	41.71 (7.16)	9–67	40.85 (7.99)	18–67
**Future validity of permit**^**e**^ (*n* = 114)	9.80 (10.37)	-2–35	9.76 (10.36)	-2–35	9.89 (10.57)	-2–35	9.57 (10.13)	-2–35	10.50 (11.25)	-2–35
**German language skills**^**f**^	2.30 (1.03)	0–4	2.51 (1.02)	0–4	1.83 (0.91)	0–3	2.39 (1.07)	0–4	2.04 (0.88)	0–4
**Number of experienced trauma types**	2.87 (2.59)	0–12	3.19 (2.74)	0–12	2.17 (2.06)	0–8	2.59 (2.34)	0–12	3.82 (3.09)	0–11
**Acculturative stress (BISS)**^**g**^										
Perceived discrimination	1.77 (0.70)	1–4	1.77 (0.66)	1–4	1.75 (0.77)	1–4	1.72 (0.67)	1–4	1.92 (0.77)	1–4
Intercultural contact stress	1.67 (0.59)	1–4	1.67 (0.55)	1–4	1.67 (0.68)	1–4	1.62 (0.58)	1–4	1.84 (0.60)	1–4
General psychosocial stress	2.13 (0.71)	1–4	2.13 (0.68)	1–4	2.13 (0.76)	1–4	2.04 (0.72)	1–4	2.42 (0.59)	1–4
Homesickness	2.44 (0.81)	1–4	2.35 (0.72)	1–4	2.64 (0.96)	1–4	2.33 (0.81)	1–4	2.79 (0.71)	1–4
Total score	1.88 (0.59)	1–4	1.88 (0.55)	1–4	1.90 (0.67)	1–4	1.82 (0.58)	1–4	2.08 (0.57)	1–4

* Totals may not sum to 100 due to rounding; ^a^ Valid values; ^b^ Multiple answers possible; ^c^ full-time, part-time or marginal employment; ^d^ Family members currently living in crisis areas; ^e^ In months; ^f^ Self-evaluation on a scale from 0 (no skills) to 4 (very good skills); ^g^ BISS = Barcelona Immigration Stress Scale; ^1^ Low somatic distress is defined as minimal or mild somatic symptoms score (PHQ-15 score < 10); ^2^ High somatic distress is defined as moderate or severe somatic symptoms score (PHQ-15 score ≥ 10)

### Health characteristics and health care utilization

From this sample, 85.3% reported at least one medical appointment within the last 12 months and 11.2% even reported more than ten appointments (Table 2). Considering only the participants with high SOD, this percentage was as high as 21.4%, compared to 7.9% for the participants with low SOD (*χ*^2^ = 6.33, *p* = 0.042, V = 0.055). In addition, 16.6% of the participating women went to a doctor more than ten times in the past year (men: 8.8%). In this respect, women consulted a doctor significantly more often than men did (*χ*^2^ = 12.33, *p* = 0.011, V = 0.399).

**Table 2:** Health characteristics of the total sample and stratification by gender and somatic distress severity (*N* = 116)

	**Total (*N*** **= 116)**		**Male (*n*** **= 80)**		**Female (*n*** **= 36)**		**Low somatic distress**^**1**^ **(*n *= 88)**		**High somatic distress**^**2**^ **(*n =*** **28)**	
	n	%^a^*	n	%^a^*	n	%^a^*	n	%^a^*	n	%^a^*
**Medical visits in the last 12 months**										
Yes	99	85.3	64	80.0	35	97.2	71	80.7	28	100.0
No	17	14.7	16	20.0	1	2.8	17	19.3	0	0.0
1–2 times	27	23.3	23	28.7	4	11.1	23	26.1	4	14.3
3–5 times	39	33.6	26	32.5	13	36.1	29	33.0	10	35.7
6–10 times	19	16.4	7	8.8	12	33.3	12	13.6	7	25.0
11–20 times	7	6.0	4	5.0	3	8.3	3	3.4	4	14.3
More than 20 times	6	5.2	3	3.8	3	8.3	4	4.5	2	7.1
**Chronic diseases**										
Yes	16	13.8	9	11.3	7	19.4	18	20.5	5	17.9
No	100	86.2	71	88.8	29	80.6	70	79.5	23	82.1
**Regular intake of medication**										
Yes	29	25.0	14	17.5	15	41.7	18	20.5	11	39.3
No	87	75.0	66	82.5	21	58.3	70	79.5	17	60.7
**Previous treatment of mental disorders**										
Yes	9	7.8	4	5.0	5	13.9	6	6.8	3	10.7
No	107	92.2	76	95.0	31	86.1	82	93.2	25	89.3
	**M** **(SD)**	**Range**	**M** **(SD)**	**Range**	**M** **(SD)**	**Range**	**M** **(SD)**	**Range**	**M** **(SD)**	**Range**
**Health status**^**b**^	2.33 (0.90)	0–4	2.46 (0.93)	0–4	2.02 (0.77)	0–4	2.49 (0.90)	0–4	1.82 (0.72)	0–4
**Mental health status**^**b**^	2.21 (0.97)	0–4	2.35 (0.96)	0–4	1.92 (0.94)	0–4	2.37 (0.93)	0–4	1.70 (0.95)	0–4

* Totals may not sum to 100 due to rounding; ^a^ Valid values; ^b^ Self-evaluation on a Scale from 0 (bad) to 4 (excellent); ^1^ Low somatic distress is defined as minimal or mild somatic symptoms score (PHQ-15 score < 10); ^2^ High somatic distress is defined as moderate or severe somatic symptoms score (PHQ-15 score ≥ 10)

Participants with high SOD rated their health status (M = 1.82, SD = 0.72) and their mental health status (M = 1.70, SD = 0.95) lower than participants with low SOD (health status: M = 2.49, SD = 0.90, *t* = 4.00, *p* ≤ 0.001, d = 0.778; mental health: M = 2.37, SD = 0.93, *t* = 3.23, *p* = 0.002, d = 0.717). A similar result was found for women in comparison to men. Women rated their health status (M = 2.02; SD = 0.77) and their mental health status (M = 1.92, SD = 0.94) significantly lower than men did (health status: M = 2.46, SD = 0.93, *t* = − 2.63, *p* = 0.010, d = 0.498; mental health: M = 2.35, SD = 0.96; *t* = − 2.23, *p* = 0.028, d = 0.451).

### Somatic symptoms

The average severity of somatic symptoms (PHQ-15 score) for the total sample was 6.35 (SD = 5.49). Almost half (49.1%) of the respondents were at risk of SOD (PHQ-15 score ≥ 6), 15.5% of the respondents showed moderate severity SOD (PHQ-15 score of 10–14), and 8.6% showed high severity SOD levels (PHQ-15 score ≥ 15). Women (M = 8.94, SD = 6.79) experienced significantly higher SOD scores than men did in the previous four weeks (M = 5.19, SD = 4.37; *t* = 3.05, *p* = 0.004, d = 0.717). Similar distinctions between women and men were found for the risk of SOD, the number of severely distressing symptoms, and the somatic symptom severity with medium effect sizes (Additional file 1). An ANCOVA with severity of somatic symptoms as the dependent variable, gender as the independent variable, and age as the covariate also revealed a significant effect of gender (F = 11.43, *p* = 0.001); the effect for age was not significant (F = 1.19, *p* = 0.278), although women had significantly higher age-adjusted mean scores than men (data not shown).

For the total sample, the most distressing symptoms (estimated as “bothered a lot”) were: Back pain (22.4%); pain in arms, legs, or joints (17.2%); nausea, flatulence or indigestion (13.8%); trouble sleeping (12.1%). Women reported back pain (25.0%), headaches (22.2%), trouble sleeping (22.2%), and pain in arms, legs, or joints (19.4%) as the most bothering symptoms. For men, these were back pain (21.3%); pain in arms, legs, or joints (16.3%); nausea, flatulence or indigestion (12.5%); trouble sleeping (7.5%). The prevalence rates for all bothering symptoms (“bothering a little” and “bothering a lot”) are depicted in Additional file 2.

The impairment in working, housekeeping, and interaction with other people caused by SOD was, on average 0.67 (SD = 0.67), indicating no or only a small impairment. Nevertheless, participants reporting high SOD (M = 1.12, SD = 0.71) had significantly more everyday problems than participants reporting low SOD severity (M = 0.53, SD = 0.59; *t* = − 3.82, *p* ≤ 0.001, d = 0.951; data not shown).

### Comorbidities between somatic distress and other common mental disorders

Of the total sample, 24.1% achieved moderate-to-severe levels of SOD (PHQ-15 score ≥ 10), 30.2% experienced clinically relevant symptoms of depression (PHQ-9 score ≥ 10), 15.5% screened positive for anxiety (GAD-7 ≥ 10), and 12.1% fulfilled the criteria of a PTSD diagnosis (ETI score). For 75.0% of the participants with moderate-to-severe levels of somatic distress, we revealed comorbid PHQ-9 levels of 10 or higher; for 46.0%, comorbid GAD-7 levels of 10 or higher; for 28.6%, comorbid clinically relevant PTSD symptoms (Additional file 3). The detailed distribution of the four severity levels of somatic symptoms by these three mental disorders is given in Additional file 4.

### Associations between somatic distress and sociodemographic, migration-specific, and health characteristics, and common mental disorders

To choose the most suitable variables for the inclusion in our regression analysis for predicting SOD, we first conducted Pearson’s and Spearman’s correlations. Among others, SOD was strongly associated with all three examined mental disorder scores: Depression (*r* = 0.70, *p* ≤ 0.001), anxiety (*r* = 0.64, *p* ≤ 0.001), and PTSD (*r* = 0.29, *p* ≤ 0.001). All of the significant correlations with SOD are presented in Additional file 5 and were included in the following multiple block-wise regression analyses. These regression analyses were conducted to examine the association between the selected sociodemographic, migration-specific, and health-related variables, and the severity of the somatic symptoms of the total sample (Table 3). Model 1 (F_(3,112)_ = 8.104, *p* ≤ 0.001) included only the sociodemographic variables (age, gender) and the number of medical visits in the past 12 months as predictors, and revealed female gender (β = − 0.21, *p* = 0.026) and a higher number of medical visits (β = 0.28, *p* = 0.003) to be significantly associated with greater somatic symptom severity. In Model 2 (F_(4,111)_ = 10.336, *p* ≤ 0.001), traumatic experiences were added and were shown to be positively associated with greater SOD severity (β = 0.32, *p* ≤ 0.000), besides female gender (β = − 0.29, *p* = 0.001) and higher number of medical visits (β = 0.23, *p* = 0.010). Adding post-migration stressors to Model 3 (F_(7,108)_ = 7.593, *p* ≤ 0.001) displayed the following significant associations: Female gender (β = 0.28, *p* = 0.014), higher number of medical visits (β = 0.22, *p* = 0.018), more different trauma types (β = 0.27, *p* = 0.003), and general psychosocial stress (β = 0.37, *p* = 0.004). The additional inclusion of common mental disorders (depression, anxiety, and PTSD symptoms) in the final model, Model 4 (F_(10,105)_ = 18.913, *p* ≤ 0.001), indicated that among these adjustments, higher number of medical visits (β = 0.15, *p* = 0.034), higher depression values (PHQ-9; β = 0.45, *p* ≤ 0.001), and higher anxiety scores (GAD-7; β = 0.31, *p* = 0.002) were significantly associated with increased somatic symptomatology. The explained variance evolved from 15.6% in Model 1, over 24.5% in Model 2 and 28.6% in Model 3 to 60.9% in Model 4.

**Table 3:** Multiple block wise linear regression analyses for the severity of somatic distress (*N* = 116)

Included variables	Significant variables ^**1**^	B^**2**^	95% CI^**3**^	SE^**4**^	β	***p***
**Model 1** (R^2^_adj._ = 0.156)^5^**:** gender, age, and number of medical visits	Gender^a^	−2.464	−4.622 to −.305	1.089	−.208	.026
	Number of medical visits	1.176	.415 to 1.938	.384	.281	.003
**Model 2** (R^2^_adj._ = 0.245)^5^**:** gender, age, number of medical visit, and traumatic experiences^6^	Gender^a^	−3.470	−5.579 to −1.361	1.065	−.293	.001
	Number of medical visits	.962	.232 to 1.691	.368	.230	.010
	Traumatic experiences	.680	.322 to .1.038	.181	.320	< .001
**Model 3** (R^2^_adj._ = 0.286)^5^**:** gender, age, number of medical visits, traumatic experiences^6^, intercultural contact stress, homesickness, and general psychosocial stress	Gender^a^	−3.419	−5.507 to −1.332	1.053	−.289	.002
	Number of medical visits	.900	.158 to 1.642	.374	.215	.018
	Traumatic experiences	.565	.121 to 5.507	.189	.266	.003
	General psychosocial stress	2.888	.926 to 4.850	.990	.370	.004
**Model 4** (R^2^_adj._ = 0.609)^5^**:** gender, age, number of medical visits, traumatic experiences^6^, intercultural contact stress, homesickness, general psychosocial stress, depression score^7^, anxiety score^8^, and PTSD score^9^	Number of medical visits	.605	.047 to 1.162	.281	.145	.034
	Anxiety score	.358	.132 to .584	.114	.308	.002
	Depression score	.451	.254 to .647	.099	.450	< .001

^1^ Significant associations with the PHQ-15 sum score; ^2^ B = regression coefficient; ^3^ CI = confidence interval; ^4^ SE = standard error; ^5^ Explanation of variance; ^6^ Number of different trauma types experienced; ^7^Measured by the PHQ-9 sum score; ^8^ Measured by the GAD-7 sum score; ^9^ Measured by the ETI sum score; ^a^ 0 = female, 1 = male

## Discussion

To the best of our knowledge, this is the first register-based study to report on the patterns of SOD in Syrian refugees with permission to reside in Germany. The results of our study highlight the great burden of somatic symptoms among Syrian refugees in Germany and the strong association between this burden and pre- and post-migration factors, and other common mental disorders. The frequent medical service utilization among somatically strained Syrian refugees should alert both policy-makers and health care providers to this highly vulnerable group.

### Health characteristics and health care utilization

Participants in the high SOD group (PHQ ≥ 10) reported significantly higher medical service utilization than participants in the low SOD group. Although the number of visits to the doctor may be partly attributed to the SOD severity and may be an indication of somatization, it must also be taken into consideration that the number of visits in the German general population (9.9 medical appointments per resident and year) is well above the international average (6.8 visits per year) [52]. This may have led to the Syrian refugees adapting to the practices of the German health system. Unfortunately, to the best of our knowledge, such a statistic does not exist for the Syrian health system, since outpatient health insurance has not been established thus far [53]. In contrast to the high percentage of health care utilization, only a few participants admitted to previous treatment of mental disorders. Among the respondents with high SOD, the percentage was only a little higher. This could possibly be related to the assumption that refugees from the Arab culture sphere prefer to be referred to medical services, rather than to psychiatric institutions, as they have limited knowledge and awareness of mental health or they fear stigmatization by the doctor, their family, or compatriots [54]. Language barriers, as well as a limited understanding of the German mental health care system, may also be reasons for the limited use of these services [15]. Given the high proportion of detected mental disorders among this refugee sample in Germany, a gap in mental health treatment becomes apparent at this point.

### Prevalence of somatic symptoms and somatic distress

The mean PHQ-15 score in our sample was lower than in comparable research among a Syrian refugee sample in Turkey [18, 32, 47]. In addition, one in four respondents of the present study experienced moderate-to-severe SOD, which is much less than in the study conducted in Turkey (43%) [18]. This discrepancy might be related to different aspects, such as better social benefits, e.g., health insurance or unemployment benefits in Germany. In Turkey, many refugees experience high levels of poverty, as well as difficulties accessing health and social services [18, 55]. However, one of the most essential differences seems to be that all of the participants in the present study were already in possession of residence permission, whereas this was not the case for the participants in the Turkish study. This difference may have a large impact on the sense of safety, on the maintained social benefits, and thus on mental health [9]. More similar prevalence rates to our results have been reported in a study among refugees that have recently arrived in Germany, using a shortened version of the PHQ-15, with 31% suffering from somatization [16, 32, 47]. In the German general population, however, the prevalence of somatization at a moderate-to-severe level is considerably lower, at 9.3% [56]. There are many possible explanations for this difference. One explanation may be that yet unknown somatic diseases based on traumatic experiences such as torture or war injuries lead to somatic complaints [15]. Another explanation for the cultural differences in SOD attributes the higher somatization prevalence of collectivistic cultures, which include Arabic countries, to the greater tendency to express psychological distress as somatic sensations, which are socially and culturally accepted [57].

Most frequently, the participants in our study reported pain in their arms, legs and joints, back, and head. This is fully consistent with the results of a systematic review among chronic pain in refugees with PTSD [58]. Furthermore, almost half of the participants were at least at risk of SOD, which is in line with previous studies among SOD in refugees [18, 32].

As an additional finding, somatically high distressed participants reported significantly more difficulties in working, housekeeping, and interaction with other people than participants with low SOD did. This indicates the high impact of SOD on the general well-being and quality of life of those affected.

### Comorbidities of somatic distress with other common mental disorders

Another finding of our study was high comorbidity between SOD and other common mental disorders. Among the participants screened positive for SOD, 75% showed comorbid depression, 46% comorbid anxiety, and 28.6% comorbid PTSD. For the comorbidity with depression, this percentage was higher than in a general refugee sample in Germany (44.9%), but for comorbid PTSD, it was much lower (60.3%) [16]. In total, our results support the existing assumption about a strong association between SOD and depression, PTSD, and anxiety among refugees [18, 32, 59].

### Risk factors for increased somatic distress

In our study, increased SOD was significantly associated with all three of the other captured mental disorders. Besides the strong association with mental disorders, we also found significant correlations of SOD with female gender, higher age, the amount of health care utilization in the past 12 months, the amount of different trauma types, and the post-migration stress factors homesickness, intercultural contact stress, and general psychosocial stress. For most of these factors, we found similar results to previous research on several refugee populations, including samples of Burmese refugees in Australia, recently arrived refugees in Germany, traumatized refugees in Switzerland, Kosovar civilian war survivors, and Syrian refugees in Turkey [16–18, 32, 33, 60]. Only information on the connection between SOD and post-migration living difficulties of refugees is widely missing in existing studies [17].

Furthermore, multiple block-wise regression analysis revealed the extent of health care utilization, anxiety symptoms, and depression symptoms to have the strongest correlation with the severity of somatic symptoms when adjusted for sociodemographic, migration-specific, and other critical variables. This analysis proved the strong association of comorbid mental disorders with SOD as their inclusion to the regression model increased the explained variance from 28.6 to 60.9%. Without the adjustment for mental disorders, female gender, amount of health care utilization, experienced traumatic events, and general psychosocial stress (e.g., financial problems or feeling alone) were shown to be significantly associated with the severity of somatic symptoms. All of these SOD associations have already been found in different samples of refugees and host countries, except for the concept of general psychosocial stress [15, 18, 23, 32]. However, parts of this construct, such as bad economic status, have already been identified as risk factors of SOD [32, 47].

One of the most striking results of our study was that the influence of post-migratory general psychosocial stress, such as loneliness, financial problems, or poor housing, on SOD seems to bewas greater than that of traumatic experiences. This finding supports previous research suggesting that post-migratory stressors are crucial factors associated with refugees’ mental health [17, 61]. Therefore, our findings suggest that the government should provide adequate living conditions after resettlement in order to alleviate the economic pressure of refugees. In the therapeutic process, it is important to pay special attention to refugees’ current living situation and acculturative adjustment process, and not only to focus on the traumatic events they have experienced in their past. Identifying and changing the psychosocial conditions is already a big step on the way to improving the quality of life and socio-cultural adaptation of refugees [62].

At this point, we would also like to highlight the identified risk factor of female gender. In our study sample, women experienced significantly higher scores of SOD than men and reported significantly more severely distressing symptoms. In addition, women consulted medical services significantly more often than men and rated their physical as well as mental health status significantly lower than men did. In this respect, women represent a highly vulnerable group among Syrian refugees and have to be specifically addressed by mental health services, municipalities, and volunteers. The following could be provided: Information material to raise awareness of mental illness, sheltered retreats, and offers to get in touch with other affected women. Special prevention programs for women, such as language courses, group therapy, vocational guidance, or women’s living groups, could help to reduce SOD and to ensure that female refugees are not left behind.

### Strengths, limitations, and further implications

To the best of our knowledge, this is the first study to explore the specific characteristics, co-occurrences, and risk factors of SOD among Syrian refugees in Germany. Especially the inclusion of acculturative stressors represents a rarity in the investigation of SOD in refugee populations. In addition, a broad range of mental disorders was taken into account using well-established, valid instruments, which allowed the calculation of different comorbidities. Another advantage was the investigation of a normal population, not a clinical sample. It should also be highlighted that the questionnaires were presented in the participants’ mother tongue and the subjects had a chance to ask questions in their mother tongue. Another strength of this study was the register-based approach, allowing us to reach the specific population of Syrian refugees with residence permission receiving social benefits systematically and in its entirety. This is a big advantage compared to other sampling methods such as the snowball sampling.

Regarding the representativeness of our sample, 64.8% of requests for asylum from Syrian refugees were made by men and 35.2% by women in the period from 2014 to 2017 [2, 63, 64]. In the present sample, 31.0% of the participants were women and 69.0% were men, which represents the gender distribution of Syrian refugees in Germany very well. The age distribution also corresponds to the overall Syrian population in Germany [2, 63, 64]. With regard to the other characteristics, however, the representativeness of our sample is clearly limited. Since our entire sample resided in a specific city in Western Germany there is a lack of generalizability to the general population of Syrian refugees in Germany. Moreover, the results are not transferable to Syrian refugees who do not receive social benefits from the government.

Another important limitation of this study is missing of information on diagnosed diseases or disabilities apart from chronic diseases. A medical examination of the participants would have been even better. The PHQ-15 screening instrument for the severity of somatic symptoms is not suitable to distinguish between symptoms that are physically or psychologically explained [65]. In this respect there was no opportunity for a medical explanation of physical symptoms, which advances the possibility that our findings overrepresent the true levels of SOD in our study population. Another problem, relating to the PHQ-15, is the different cut-off scores used in previous research for detecting SOD or somatization (e.g., PHQ-15 score ≥ 6 or ≥ 10, or the presence of at least three severely bothering somatic symptoms) [18, 32, 45]. This complicated the comparability among study results and should be standardized in the future. The fact that different terminology and definitions exist for the construct measured by the PHQ-15 questionnaire also makes a comparison difficult (e.g., SOD, somatic symptom severity, somatization, somatic symptom burden, somatic symptom disorder, or somatoform disorder).

This study followed a cross-sectional design that did not allow for drawing causal conclusions concerning the influence of the measured variables on SOD. Longitudinal data are needed to assess the temporal relationships between SOD and associated factors, such as acculturative stress. The relatively small sample size was well suited to detect medium-to-large statistical effects, but may have impacted the Type II error so that small statistical significant effects could not be detected because of the limited power of the statistical tests. It also has to be mentioned that all of the data were assessed using self-report questionnaires, which are usually a source of bias.

Despite the limitations and the implications for future research mentioned above, our findings have further implications for German policy and health care practice. Key players in health care systems and political authorities need to be aware of the strong links between physical and mental health disorders. The mental health problems underlying physical symptoms can lead to chronicity, resulting in social withdrawal, lack of ability to integrate, and increased costs of care [15, 66]. Therefore, health care professionals should place importance on the differential diagnosis of medically unexplained physical symptoms in refugees. Instead of only focusing on treating specific somatic symptoms, the possibility of underlying mental disorders, traumatization, or post-migration stressors should be considered. This would contribute to minimizing the risk of misdiagnoses. Due to specific cultural differences and language difficulties, trained interpreters must be provided for medical examinations. In summation, there is strong evidence that culturally sensitive and adapted psychological approaches can help in the treatment of SODs and related mental disorders to increase individual well-being and to reduce the need for medical service utilization [67].

## Conclusions

In addition to previous research highlighting the high levels of PTSD, depression, and anxiety, our study revealed the high prevalence rates of Syrian refugees with residence to reside in Germany suffering from SOD. While anyone can suffer from SOD, health care professionals should be particularly vigilant to women, those having experienced traumatic events, those reporting psychosocial stress, and those with more severe depression and anxiety symptom scores. Furthermore, in our sample, Syrian refugees bothered by moderate-to-severe SOD were more likely to frequent health care utilization. Appropriate prevention and treatment of SOD and its related mental health disorders would therefore not only improve the quality of life of those affected, but also reduce related health care costs.

## Supplementary Information

**Additional file 1 **Mean scores and frequencies of somatic distress among the total sample and stratification by gender (*N* = 116).

**Additional file 2 **Somatic symptoms causing distress among the total sample and stratification by gender (*N* = 116).

**Additional file 3 **Comorbidities with depression, anxiety, and PTSD among the participants with moderate-to-severe levels of somatic distress (*N* = 28).

**Additional file 4 **The severity of somatic distress stratified by mental disorders common among refugees, for the total sample (*N* = 116).

**Additional file 5 **Correlation coefficients between somatic distress, sociodemographic, pre- and post-migration variables, and common mental disorders (*N* = 116).

## Data Availability

The dataset analyzed during the current study is available from the corresponding author on reasonable request.

## Abbreviations

SOD: Somatic distress
PTSD: Post-traumatic stress disorder
GAD: Generalized anxiety disorder
PHQ-9: Patient Health Questionnaire – depression module
PHQ-15: Patient Health Questionnaire – somatic symptom module
GAD-7: Generalized Anxiety Disorder Scale
B: Regression coefficient
CI: Confidence interval
SE: Standard error
