# Supplementary material for: Somatic distress among Syrian refugees with residence permission in Germany: analysis of a cross-sectional register-based study
Source: BMC Public Health. 2021 May 12;21:896. doi: 10.1186/s12889-021-10731-x (PMC8114491; doi:10.1186/s12889-021-10731-x)
Supplement: Supplementary file 1 — Additional file 1 Mean scores and frequencies of somatic distress among the total sample and stratification by gender (N = 116). [file 12889_2021_10731_MOESM1_ESM.docx]

**Somatic distress among Syrian refugees with residence-permission in Germany: analysis of a cross-sectional register-based study**

Andrea Borho^1,^*, Eva Morawa^1^, Gregor Martin Schmitt^2^, Yesim Erim^1^

^1^Department of Psychosomatic Medicine and Psychotherapy, Friedrich-Alexander University Erlangen-Nürnberg (FAU), Erlangen, Germany ^2^Erlangen City Council, Job Center, Erlangen, Germany

* Corresponding author: andrea.borho@uk-erlangen.de; Tel.: +49-9131-85-44321

**Additional file 1.** Mean scores and frequencies of somatic distress among the total sample and stratification by gender (*N* = 116)

|  | **Total (*N* = 116)** | **Male (*n* = 80)** | **Female (*n* = 36)** | **Comparison** | | |
| --- | --- | --- | --- | --- | --- | --- |
|  | **M (SD)** | **M (SD)** | **M (SD)** | ***t/ χ²*** | ***p*** | **d** |
| **PHQ-15 score** | 6.35 (5.49) | 5.19 (4.37) | 8.94 (6.79) | 3.05^5^ | .004 | .717^8^ |
| **PHQ-15* score^1^** | 6.11 (5.23) | 5.10 (4.18) | 8.36 (6.55) | 2.75^5^ | .008 | .648^8^ |
| **Number of severely distressing symptoms^2^** | 1.20 (2.05) | 0.80 (1.36) | 2.08 (2.90) | 2.53^5^ | .015 | .651^8^ |
|  | **n (%^a, b^)** | **n (%^a, b^)** | **n (%^a, b^)** |  |  |  |
| **Risk of somatic distress^3^** |  |  |  |  |  |  |
| Yes  No | 57 (49.1)  59 (50.9) | 34 (42.5)  46 (57.5) | 23 (63.9)  13 (36.1) | 4.54^6^ | .033 | .198^9^ |
| **Somatic distress severity^4^** |  |  |  |  |  |  |
| Minimal  Mild  Moderate  Severe | 52 (44.8)  36 (31.0)  18 (15.5)  10 (8.6) | 40 (50.0)  28 (35.0)  9 (11.3)  3 (3.8) | 12 (33.3)  8 (22.2)  9 (25.0)  7 (19.4) | 12.06^7^ | .001 | .334^9^ |

^1^ PHQ-15 score without the menstrual cramps or problems item; ^2^ Mean number of somatic symptoms rated as “bothered a lot”; ^3^ A PHQ-15 score of 6 or more classified respondents as being at risk of somatic distress; ^4^ Severity categories based on PHQ-15 score: minimal (0-4), mild (5-9), moderate (10-14) and severe (≥ 15); ^5^ Independent t-test; ^6^ Chi-squared test; ^7^ Fisher’s exact test; ^8^ Cohen’s d (Hedges’ g) ^9^ Cramer-V; ^a^ Valid values; ^b^ Totals may not sum to 100 due to rounding
