# Supplementary material for: Somatic distress among Syrian refugees with residence permission in Germany: analysis of a cross-sectional register-based study
Source: BMC Public Health. 2021 May 12;21:896. doi: 10.1186/s12889-021-10731-x (PMC8114491; doi:10.1186/s12889-021-10731-x)
Supplement: Supplementary file 2 — Additional file 2 Somatic symptoms causing distress among the total sample and stratification by gender (N = 116). [file 12889_2021_10731_MOESM2_ESM.docx]

**Somatic distress among Syrian refugees with residence-permission in Germany: analysis of a cross-sectional register-based study**

Andrea Borho^1,^*, Eva Morawa^1^, Gregor Martin Schmitt^2^, Yesim Erim^1^

^1^Department of Psychosomatic Medicine and Psychotherapy, Friedrich-Alexander University Erlangen-Nürnberg (FAU), Erlangen, Germany ^2^Erlangen City Council, Job Center, Erlangen, Germany

* Corresponding author: andrea.borho@uk-erlangen.de; Tel.: +49-9131-85-44321

**Additional file 2.** Somatic symptoms causing distress among the total sample and stratification by gender (*N* = 116)

|  | **Total (*N* = 116)** | **Male (*n* = 80)** | **Female (*n* = 36)** |
| --- | --- | --- | --- |
|  | **n (%^a, b^)** | **n (%^a, b^)** | **n (%^a, b^)** |
| **PHQ-15 symptoms^1^** |  |  |  |
| Pain in arms, legs or joints  Back pain  Feeling tired, having low energy  Trouble sleeping  Headaches  Shortness of breath  Nausea, flatulence or indigestion  Constipation, nervous bowel, or diarrhea  Chest pain  Heart pounding or racing  Stomach pain  Dizziness  Pain or problems during sex  Fainting spells  Menstrual cramps or problems^2^ | 65 (56.0) 64 (55.2)  60 (51.7)  59 (50.9)  55 (47.4)  48 (41.3)  40 (34.5)  37 (31.9)  35 (30.2)  35 (30.1)  31 (26.8)  26 (22.4)  13 (11.2)  7 (6.1) | 42 (52.6)  42 (52.6)  36 (45.0)  38 (47.5)  36 (45.0)  27 (33.8)  23 (28.8)  22 (27.6)  18 (22.5)  19 (23.8)  18 (22.6)  14 (17.5)  8 (10.1)  3 (3.8) | 23 (63.8)  22 (61.1)  24 (66.7)  21 (58.3)  19 (52.8)  21 (58.4)  17 (47.3)  15 (41.7)  17 (47.3)  16 (44.5)  13 (36.1)  12 (33.4)  5 (13.9)  4 (11.1)  17 (47.2) |

^1^ Somatic symptoms rated as bothering (“bothered a little” or “bothered a lot”); ^2^ Calculated for female participants only; ^a^ Valid values; ^b^ Totals may not sum to 100 due to rounding
