# Supplementary material for: Somatic distress among Syrian refugees with residence permission in Germany: analysis of a cross-sectional register-based study
Source: BMC Public Health. 2021 May 12;21:896. doi: 10.1186/s12889-021-10731-x (PMC8114491; doi:10.1186/s12889-021-10731-x)
Supplement: Supplementary file 3 — Additional file 3 Comorbidities with depression, anxiety, and PTSD among the participants with moderate-to-severe levels of somatic distress (N = 28). [file 12889_2021_10731_MOESM3_ESM.docx]

**Somatic distress among Syrian refugees with residence-permission in Germany: analysis of a cross-sectional register-based study**

Andrea Borho^1,^*, Eva Morawa^1^, Gregor Martin Schmitt^2^, Yesim Erim^1^

^1^Department of Psychosomatic Medicine and Psychotherapy, Friedrich-Alexander University Erlangen-Nürnberg (FAU), Erlangen, Germany ^2^Erlangen City Council, Job Center, Erlangen, Germany

* Corresponding author: andrea.borho@uk-erlangen.de; Tel.: +49-9131-85-44321

**Additional file 3.** Comorbidities with depression, anxiety, and PTSD among the participants with moderate-to-severe levels of somatic distress (*N* = 28)*

* The presented 95% confidence intervals were constructed by performing empirical bootstrap resampling on the basis of 100000 resamples.
