# Supplementary material for: Somatic distress among Syrian refugees with residence permission in Germany: analysis of a cross-sectional register-based study
Source: BMC Public Health. 2021 May 12;21:896. doi: 10.1186/s12889-021-10731-x (PMC8114491; doi:10.1186/s12889-021-10731-x)
Supplement: Supplementary file 4 — Additional file 4 The severity of somatic distress stratified by mental disorders common among refugees, for the total sample (N = 116). [file 12889_2021_10731_MOESM4_ESM.docx]

**Somatic distress among Syrian refugees with residence-permission in Germany: analysis of a cross-sectional register-based study**

Andrea Borho^1,^*, Eva Morawa^1^, Gregor Martin Schmitt^2^, Yesim Erim^1^

^1^Department of Psychosomatic Medicine and Psychotherapy, Friedrich-Alexander University Erlangen-Nürnberg (FAU), Erlangen, Germany ^2^Erlangen City Council, Job Center, Erlangen, Germany

* Corresponding author: andrea.borho@uk-erlangen.de; Tel.: +49-9131-85-44321

**Additional file 4.** The severity of somatic distress stratified by mental disorders common among refugees, for the total sample (*N* = 116)

|  | **Severity of somatic distress (n, %)** | | | |
| --- | --- | --- | --- | --- |
|  | Minimal | Mild | Moderate | Severe |
| **Depression (PHQ-9 score)^a^** |  |  |  |  |
| No (<10) | 49 (42.2) | 25 (21.6) | 6 (5.2) | 1 (0.9) |
| Yes (≥ 10) | 3 (2.6) | 11 (9.5) | 12 (10.3) | 9 (7.8) |
| **Anxiety (GAD-7 score)^b^** |  |  |  |  |
| No (<10) | 52 (44.8) | 31 (26.7) | 14 (12.1) | 1 (0.9) |
| Yes (≥ 10) | 0 (0.0) | 5 (4.3) | 4 (3.4) | 9 (7.8) |
| **PTSD (ETI score)^c^** |  |  |  |  |
| No | 31 (26.7) | 23 (19.8) | 11 (9.5) | 3 (2.6) |
| Yes | 2 (1.7) | 4 (3.4) | 3 (2.6) | 5 (4.3) |

^a^ PHQ-9, Patient Health Questionnaire – Depression Module total score; ^b^ GAD-7, Generalized Anxiety Disorder total score; ^c^ ETI, Essen Trauma Inventory: sum score calculated only for participants with at least one traumatic experience on the basis of the three subscales (intrusion, hyperarousal and avoidance) that are relevant for the cut-off for a PTSD diagnosis
