# Supplementary material for: Somatic distress among Syrian refugees with residence permission in Germany: analysis of a cross-sectional register-based study
Source: BMC Public Health. 2021 May 12;21:896. doi: 10.1186/s12889-021-10731-x (PMC8114491; doi:10.1186/s12889-021-10731-x)
Supplement: Supplementary file 5 — Additional file 5 Correlation coefficients between somatic distress, sociodemographic, pre- and post-migration variables, and common mental disorders (N = 116). [file 12889_2021_10731_MOESM5_ESM.docx]

**Somatic distress among Syrian refugees with residence-permission in Germany: analysis of a cross-sectional register-based study**

Andrea Borho^1,^*, Eva Morawa^1^, Gregor Martin Schmitt^2^, Yesim Erim^1^

^1^Department of Psychosomatic Medicine and Psychotherapy, Friedrich-Alexander University Erlangen-Nürnberg (FAU), Erlangen, Germany ^2^Erlangen City Council, Job Center, Erlangen, Germany

* Corresponding author: andrea.borho@uk-erlangen.de; Tel.: +49-9131-85-44321

|  | **Somatic distress** | **Gender** | **Age** | **Medical visits** | **Traumatic experiences** | **Intercultural contact stress** | **Homesickness** | **General psychosocial stress** | **Depression** | **Anxiety** | **PTSD** |
| --- | --- | --- | --- | --- | --- | --- | --- | --- | --- | --- | --- |
| **Somatic distress** | xx |  |  |  |  |  |  |  |  |  |  |
| **Gender^1^** | -.263** | xx |  |  |  |  |  |  |  |  |  |
| **Age** | .204* | -.193* | xx |  |  |  |  |  |  |  |  |
| **Number of medical visits** | .347** | -.225* | -.009 | xx |  |  |  |  |  |  |  |
| **Traumatic experiences^2^** | .310** | .184* | .157 | .030 | xx |  |  |  |  |  |  |
| **Intercultural contact stress** | .223* | .045 | -.137 | .177 | .209* | xx |  |  |  |  |  |
| **Homesickness** | .294** | -.167 | .154 | .188* | .351** | .415** | xx |  |  |  |  |
| **General psychosocial stress** | .339** | .002 | -.113 | .173 | .288** | .715** | .557** | xx |  |  |  |
| **Depression** | .703** | -.170 | .007 | .295** | .419** | .375** | .264** | .455** | xx |  |  |
| **Anxiety** | .642** | -.205* | .049 | .345** | .341** | .307** | .320** | .444** | .702** | xx |  |
| **PTSD** | .288** | -.152 | .195 | .138 | .415** | .249* | .346** | .309** | .364** | .435** | xx |

**Additional file 5.** Correlation coefficients between somatic distress, sociodemographic, pre- and post-migration variables, and common mental disorders (*N* = 116)

* *p* < .05; ** *p* ≤ .001; ^1^ gender: 0 = female; 1 = male; ^2^ Number of different trauma types experienced; The following variables were also correlated with somatic distress severity but without significant association: years of education, duration of residence in Germany, future validity of residence permission, German language skills, family in crisis areas, chronic diseases, and perceived discrimination.
